# Impact of a Multifaceted Antimicrobial Stewardship Intervention in a Primary Health Care Area: A Quasi-Experimental Study

**DOI:** 10.3389/fphar.2020.00398

**Published:** 2020-04-02

**Authors:** Pablo March-López, Rosa Madridejos, Rosa Tomas, Lucía Boix, Paula Arcenillas, Lucía Gómez, Emma Padilla, Mariona Xercavins, Laura Martinez, Montserrat Riera, Cristina Badia, Jordi Nicolás, Esther Calbo

**Affiliations:** ^1^Hospital Pharmacy, Pharmacy Department, Hospital Universitari Mútua Terrassa, Barcelona, Spain; ^2^Primary Care Pharmacy, Pharmacy Department, Hospital Universitari Mútua Terrassa, Barcelona, Spain; ^3^Medicine Department, Universitat Internacional de Catalunya, Barcelona, Spain; ^4^Infectious Diseases Unit, Department of Internal Medicine, Hospital Universitari Mútua Terrassa, Barcelona, Spain; ^5^Microbiology Department, Centre d’analítiques Terrassa (Catlab), Barcelona, Spain; ^6^Infection Control Nurse, Hospital Universitari Mútua Terrassa, Barcelona, Spain; ^7^Clinical Pharmacy Department, Universitat de Barcelona, Barcelona, Spain

**Keywords:** antimicrobial-stewardship, outpatient, antibiotic-consumption, Centers for Disease Control and Prevention, Primary-Health-Care

## Abstract

The aim of the study was to evaluate the impact of a multifaceted antimicrobial stewardship intervention on antibiotic consumption in a primary health care (PHC) area in Spain. Quasi-experimental study conducted in a PHC area with nine PHC centers, a 400-bed acute care teaching hospital, and 18 nursing homes serving a population of 260,561. The intervention was based on the 2016 CDC Core Elements of Outpatient Antibiotic Stewardship publication and targeted 130 PHC physicians, 41 PHC pediatricians, 19 emergency physicians, and 18 nursing home physicians. The components were commitment, actions for improving antibiotic prescribing, tracking and feedback, and education and experience. The primary outcome was overall antibiotic consumption. Secondary outcomes were consumption of antibiotics to treat pharyngotonsillitis, acute otitis media, acute sinusitis, acute bronchitis, and urinary tract infection (UTI), percentage of patients treated with specific antibiotics, and dispensing costs. Consumption was measured in defined daily doses per 1,000 inhabitants per day (DID) and compared pre- and postintervention (2016 *vs.* 2018). Overall antibiotic consumption decreased from 16.01 to 13.31 DID (−16.85%). Consumption of amoxicillin/clavulanic acid and quinolones decreased from 6.04 to 4.72 DID (−21.88%) and 1.64 to 1.23 DID (−25.06%), respectively. The percentage of patients treated with antibiotics decreased from 26.99 to 22.41%. The intervention resulted in cost savings of €72,673. Use of antibiotics to treat pharyngotonsillitis, UTI, and acute otitis media, sinusitis, and bronchitis decreased significantly. Our antimicrobial stewardship program led to a decrease in antibiotic consumption and significantly improved the use of antibiotics for the most prevalent PHC infections.

## Introduction

Antimicrobial resistance is growing and is now recognized as a major global health threat (World Health Organization, 2014). It is responsible for an estimated 700,000 deaths a year (25,000 in the European Union) and is associated with annual expenditure and productivity losses amounting to €1.5 billion (European Centre for Disease Prevention and Control, European Medicines Agency, 2009). If the current trend is not reversed, estimates indicate that by 2050 antimicrobial resistance could result in 10 million deaths each year and economic losses of up to $100 trillion dollars (O’Neill, 2016).

Spain is one of the highest consumers of antibiotics in the developed world. Total antibiotic consumption in 2016 was 32.9 defined daily doses per 1,000 inhabitants per (DID) (European Centre for Disease Prevention and Control, 2018). compared with 22.8 DID for the European Union and 10.4 for the Netherlands. In the public PHC setting, consumption increased significantly from 19.70 DID in 2012 to 23.00 DID in 2016 (European Centre for Disease Prevention and Control, 2017) (overall increase of 12.69%).

Outpatient antibiotic use has been clearly correlated with antibiotic resistance in specific geographic areas, and it may even influence resistance rates in hospital settings (Seppälä et al., 1997; Goossens et al., 2005). Between 2001 and 2016, *Escherichia coli* resistance to third-generation cephalosporins increased from 0.6 to 15.0% in Spain and from 0.6 to 6.4% in the Netherlands (Susceptibility of Escherichia coli Isolates to 3rd gen. cephalosporins in Spain, Netherlands, Estonia, Latvia, 1998 – 2014; European Centre for Disease Prevention and Control, 2017).

The 2016 Centers for Disease Control and Prevention (CDC) publication on outpatient antibiotic stewardship describes four core elements for assessing and improving antibiotic prescribing in outpatient settings: 1) commitment, 2) action for policy and practice, 3) tracking and reporting, 4) and education and expertise (Sanchez et al., 2016). In 2014, the Spanish Ministry of Health published a 5-year national strategic plan for combating antimicrobial resistance (Strategic Action Plan to reduce the risk of selection and dissemination of antibiotic resistance). The main objective was to reduce the risk of selection and spread of antibiotic resistance, but the document also highlighted the importance of promoting actions to improve antibiotic prescribing (Strategic Action Plan to reduce the risk of selection and dissemination of antibiotic resistance). In line with this strategic plan and with the recommendations of the CDC (Strategic Action Plan to reduce the risk of selection and dissemination of antibiotic resistance), we implemented a multifaceted, real-world, antimicrobial stewardship program (ASP) aimed at reducing overall and specific use of antibiotics in a primary health care (PHC) area in Spain. No previous interventions had been conducted in this area.

## Material and Methods

### Design and Setting

We performed a quasi-experimental study to evaluate the program. Data were collected in three phases: a baseline phase (January–December 2016), an intervention phase (January–December 2017), and a sustainability phase (January–December 2018).

The setting for the intervention was a PHC area in the province of Barcelona that serves a population of 260,657 inhabitants and has nine PHC centers, a 400-bed acute care teaching hospital managed by the same health organization, together with 18 nursing homes. The Spanish national health care system offers universal coverage to a population of 46.4 million inhabitants (2016) (Catálogo de Centros de Atención Primaria del SNS y de Atención Urgente Extrahospitalaria Madrid (Ministerio de Sanidad Consumo y Bienester Social)). The country has 17 autonomous communities, each of which is responsible for offering regional health care services with oversight from the Spanish Ministry of Health. The country has approximately 13,000 PHC centers and 457 hospitals, and each regional health authority collects data on all prescriptions issued within the public health care system (Catálogo de Centros de Atención Primaria del SNS y de Atención Urgente Extrahospitalaria Madrid (Ministerio de Sanidad Consumo y Bienester Social)).

### Participants

The ASP targeted 130 PHC physicians, 41 PHC pediatricians, 19 emergency physicians, and 18 nursing home physicians. It also included an educational campaign targeting patients. All physicians and patients were included, no sampling was made.

### Intervention

All components of the ASP were implemented simultaneously by the PHC antimicrobial stewardship team set up in 2016 to work in coordination with the hospital-based team formed 3 years earlier. The PHC team is composed of 10 PHC physicians, four PHC pediatricians, three clinical pharmacists, an infectious disease specialist, and a microbiologist.

The components of the intervention, all evidence-based and detailed below, were selected to align with the CDC’s four core elements of outpatient antibiotic stewardship (Sanchez et al., 2016).

- *Commitment to the ASP*. Thirty minute face-to-face sessions were organized to present the ASP to all relevant stakeholders in the PHC area (management, center directors, general physicians, pediatricians, and emergency physicians). Twenty-five sessions were held to ensure 100% coverage and 150 poster reminders were distributed among the physicians’ offices and the emergency areas of the participating PHC centers. In case that a new physician was hired during the intervention or sustainability phase the AS Team presented the project and provided the educational material through a face-to-face meeting. A member of the antimicrobial stewardship team visited each of the nursing homes to explain the intervention to the physicians working there and provide them with educational material on good antibiotic prescribing practices. All care providers were asked to commit to the ASP but were not required to sign any documents to this effect. No financial incentives were offered.
- *Actions for improving antibiotic prescribing*. All PHC centers were supplied with a rapid test for detecting *Streptococcus pyogenes* and an accompanying protocol based on the Centor clinical scoring scale (modified McIsaac score) to ensure correct use of the test (Fine et al., 2012).
- *Tracking and feedback*. Every 3 months, physicians received an updated anonymized report containing qualitative and quantitative indicators on antibiotic consumption in their PHC center (see **Supplementary data** for sample report). The report contained a comparison with the same period from the previous year and also allowed physicians to compare their performance with that of other physicians and with the center overall. The data were presented as part of an interactive clinical workshop.
- *Education and experience*. The antimicrobial stewardship team also prepared a set of local guidelines on antibiotic usage and preferred regimens for adult and pediatric patients. These guidelines were distributed to physicians and were also accessible through the centralized health organization website. A total of 500 printed guidelines were distributed. In addition, 60 interactive workshops of 45 min duration were held in all the participating centers to discuss clinical cases involving respiratory tract, urinary tract, and skin and soft tissue infections. The average participant rate in the face-to-face session was 66% (125 physicians) and 85% participated in at least one session. All physicians received the workshop material by email. Over 600 project-related emails were sent. Finally, purpose-designed educational leaflets and posters (n = 150) on safe antibiotic use targeting patients were placed in waiting rooms and common areas.

### Outcomes

Antibiotic consumption was measured in DID day calculated with drug packages in all cases. The primary outcome was overall antibiotic consumption. Secondary outcomes were i) consumption of amoxicillin (ATC: J01CA04), amoxicillin/clavulanic acid (ATC: J01CR02), macrolides (ATC: J01FA, including: J01FA02 spiramycin, J01FA07 josamycin, J01FA09 clarithromycin and J01FA10 azithromycin), and quinolones (ATC: J01M, including: J01MA02 ciprofloxacin, J01MA06 norfloxacin, J01MA12 levofloxacin and J01MA14 moxifloxacin) (the main antibiotics prescribed in PHC); ii) consumption of narrow-spectrum antibiotics (penicillins with extended spectrum [ATC: J01CA, including: J01CA01 ampicillin and J01CA04 amoxicillin], beta-lactamase sensitive penicillins [ATC: J01CE, including: J01CE01 benzylpenicillin, J01CE02 phenoxymethylpenicillin, J01CE08 benzathine benzyl penicillin, J01CE10 benzathine phenoxymethylpenicillin, J01CE30 combinations], beta-lactamase resistant penicillins [ATC: J01CF, including: J01CF02 cloxacillin], and fosfomycin [J01XX01]); Antibiotics with less than 0.00 DID was considered negligible. iii) percentage of patients treated with antibiotics for common PHC conditions (pharyngotonsillitis, acute otitis media, acute sinusitis, acute bronchitis, and urinary tract infections); iv) direct costs associated with antibiotic consumption; v) unique patients prescribed at least one course of antibiotics; and vi) antibiotic consumption in nursing homes measured as defined daily doses per 100 bed days (DDBs). Indicators were measured at baseline and during the intervention and sustainability phases.

### Data Collection

Data were extracted from the patients’ electronic medical records throughout the study period. Patient contacts were traced by checking records for ICD-9 codes for relevant infections and ATC codes for antibiotic prescriptions. In order to calculate the economic indicator, the reference prices for drugs stablished by the Ministry of Health, Consumer Affairs and Social Welfare of Spain were applied (Real Decreto 177/2014, de 21 de marzo, por el que se regula el sistema de precios de referencia y de agrupaciones homogéneas de medicamentos en el Sistema Nacional de Salud, y determinados sistemas de información en materia de financiación y precios de los medicamentos y productos sanitarios).

### Statistical Analysis

A descriptive analysis was performed for each outcome indicator using data collected before and after the intervention (baseline/2016 *vs.* sustainability phase/2018). Proportions and SDs were used for the descriptive statistics. Proportions of categorical variables in two or more independent groups were compared using the Chi-square test. P-values ≤ 0.05 were considered statistically significant. When required, statistical trend analyses were carried out to evaluate the significance of temporal variations. All endpoint definitions and statistical methods were predefined in a statistical analysis plan before evaluation of the data. Analyses were performed using Stata 13 (Stata Corporation, College Station, TX, USA).

The project was approved by the Mútua Terrassa Research Ethics Committee; informed consent was not required according to institutional and national guidelines.

## Results

### Demographic Characteristics

Data were analyzed for 260,561 patients, 50.89% of whom were female. The mean (SD) age was 40.85 (22.81) years; 20.98% of the patients were pediatric (0–14 years old) and 16.07% were aged 65 years or older. The study population included 1,964 nursing home residents with a mean age of 79.99 (8.22) years. A mean of 181,863 unique-patient visits per year was registered over the study period.

### Quantitative Indicators

Overall antibiotic consumption decreased by 16.85% (16.01 to 13.31 DID) between 2016 (baseline) and 2018 (sustainability period). It decreased by 16.94% in the adult population (17.34 to 14.41 DID) and by 19.22% in the pediatric population (10.05 to 8.12 DID). The percentage of patients treated with antibiotics also decreased, from 26.99% in 2016 to 22.41% in 2018 (−4.57%; p < 0.05). Overall antibiotic consumption decreased in both adults and children but the largest decrease (26.00%) was observed in nursing home residents (7.74 DDB in 2016 to 5.72 DDB in 2018).

On analyzing consumption of specific antibiotics, significant reductions were observed for amoxicillin (4.75 to 4.08 DID; −14.12%), amoxicillin/clavulanic acid (6.04 to 4.72 DID; −21.88%), quinolones (1.64 to 1.23 DID; −25.06%), and macrolides (1.73 to 1.43 DID; −17.18%). Consumption of narrow-spectrum antibiotics increased from 31.32 to 32.35% (+1.04%; p < 0.05). The overall results of the ASP are summarized in **Tables 1** and **2**.

### Qualitative Indicators

The percentage of adults diagnosed with pharyngotonsillitis and treated with antibiotics decreased significantly from 64.81% in 2016 to 54.38% in 2018 (−10.43%; p < 0.05). The corresponding reduction for children was 12.68% (56.53 to 43.85%, p < 0.05). In both adult and pediatric patients, there was a significant increase in the use of amoxicillin to treat pharyngotonsillitis and a decrease in the use of amoxicillin/clavulanic acid and macrolides. Further information over qualitative data for the treatment of pharyngotonsillitis in the three periods of study is shown in **Table 3**.

The proportion of patients with acute otitis media treated with antibiotics also decreased: from 58.26 to 49.53% (−8.72%; p < 0.05) in the case of adults and from 72.37 to 68.09% (−4.28%, p < 0.05) in the case of children. As with pharyngotonsillitis, there was a significant increase in the use of amoxicillin and a decrease in that of amoxicillin/clavulanic acid and macrolides in the adult and pediatric populations. Further information over qualitative data for the treatment of acute otitis media in the three periods of study is shown in **Table 4**.

Nonsignificant changes were observed for the percentage of patients with urinary tract infections treated with antibiotics. Adults increased from 70.94 to 71.40% (0.46%; p = 0.69), and children decreased from 51.15 to 49.28% (−1.87%; p = 0.51). In adults, the use of fosfomycin and cefuroxime increased from 39.58 to 45.36% and 26.76 to 29.93% while that of amoxicillin/clavulanic acid and quinolones decreased from 6.15 to 4.24% and 27.52 to 20.47%, respectively. Further information over qualitative data for the treatment of UTI in the three periods of study is shown in **Table 2** of the **Supplementary Material**.

Finally, there was a significant decrease in the use of antibiotics to treat acute bronchitis in both adults (70.06 to 58.65%; −11.42%; p < 0.05) and children (29.19 to 22.78%; −2.14%; p < 0.05). In the case of acute sinusitis, antibiotic use fell significantly in adults (77.50 to 68.12%; −9.38%; p < 0.05) but increased, albeit insignificantly, in children (from 80.64 to 81.27%; 0.64%; p = 0.50). Further information over the percentage of patients treated with antibiotic with diagnosis of acute sinusitis and acute bronchitis is shown in **Table 3** of the **Supplementary Material**.

### Economic Indicators

Total spending on antibiotics fell from €905,700.76 during the baseline phase to €793,765.89 during the sustainability phase (overall saving of €111,934.87), reductions were observed in the total spending of Amoxicillin/Clavulanic (overall saving of €45,935.76) and Quinolones (overall saving of €43,475.17) in correlation with the decrease in DID. Economic data for the baseline, intervention, and sustainability phases are shown in **Table 4** of the **Supplementary Material**.

## Discussion

The findings of this quasi-experimental study show that a multifaceted ASP reduced overall antibiotic consumption and costs in a PHC area. Our study supports previous reports that behavioral and educational interventions lead by pharmacists can improve antibiotic prescribing practices (Grijalva et al., 2009; Hürlimann et al., 2015; Meeker et al., 2016). In 2017 Mohsen Ali Murshid et al. developed a theoretical model of prescribing decision based on the review of previous theoretical models. The authors suggested the possible relationship between various variables related to physician decision prescribing such as pharmacy–physician collaboration or patient’s expectations (Murshid and Mohaidin, 2017).

The primary aim of our intervention was to reduce antibiotic consumption and we achieved an absolute reduction of 16.85% between 2016 and 2018. The corresponding reduction in antibiotic prescriptions in the Spanish national health care system was 4.99% (European Centre for Disease Prevention and Control, 2018), which is slightly lower than the rate of 6.53% reported for Sweden, which has one of the lowest antibiotic consumption rates in Europe (Meeker et al., 2016).

Use of narrow-spectrum antibiotics in our PHC area increased by 1.04%, which is just slightly higher than the increase of 0.44% reported for the Spanish public health care system for the same period (European Centre for Disease Prevention and Control, 2018). Use of amoxicillin/clavulanic acid in our area fell to 4.08 DID in 2018. This compares with 7.57 DID for Spain as a whole and 0.31 for Sweden. The respective figures for quinolones were 1.23 DID for our PHC area, 2.71 DID for Spain, and 0.60 DID for Sweden. Overall, 22.69% of our population was treated with at least one course of antibiotics compared with 17.4% of the Swedish population (Swedres-Svarm, 2018). It should be recalled that our figures do not include prescriptions issued in private practice. Differences in antibiotic consumption between both countries are multifactorial, but we believe one of the key elements is that Sweden has been working in a National Strategic Program against antibiotic resistance (known as Strama) (Swedish work on containment of antibiotic resistance, 2014) since 1995 with multifaceted interventions and multiprofessional teams on both national and local levels. In Spain the first National Strategic Program against antibiotic resistance was published in 2014, 19 years later. The larger reduction achieved in our PHC area can probably be explained by the fact that local strategies tend to have a greater impact than national ones, national strategies should promote and coordinate local ASP.

Most antimicrobial stewardship interventions to date have targeted single conditions (Ranji et al., 2008; Vellinga et al., 2016). In a systematic review of interventions to reduce unnecessary antibiotic prescribing, 38 of the 43 studies analyzed addressed acute respiratory infections only (Ranji et al., 2008). We decided to adopt a more comprehensive approach, as antibiotic consumption in our PHC area had increased by 13.78% between 2012 (14.00 DID) and 2016 (16.01 DID). We targeted pharyngotonsillitis, acute otitis media, acute sinusitis, acute bronchitis, and urinary tract infections as these are the most common diagnoses for which antibiotics are prescribed in our setting and they have also been associated with overprescribing (Hersh et al., 2011; Lemiengre et al., 2012; Hürlimann et al., 2015; Venekamp et al., 2015; Tystrup et al., 2016; Smith et al., 2017; Hansen et al., 2018).

Overall, 68.12% of patients with acute sinusitis were treated with antibiotics during the sustainability phase of our study. This is slightly higher than the rate of 60.6% reported for Sweden in 2013 (Tystrup et al., 2016) and considerably lower than that of 80% reported for the United States (Hansen et al., 2018). The corresponding rate for acute otitis media infections treated with antibiotics in our area was 49.53%, which contrasts with the respective rates of 45.8 and 74.9% reported for the Netherlands (Van den Broek d’Obrenan et al., 2014) and Sweden (Tystrup et al., 2016). Urinary tract infections were treated with antibiotics in 71.40% of the patients in our study *versus* 60.4% of those in the Netherlands (Van den Broek d’Obrenan et al., 2014). The antibiotic prescribing rate for sore throat (including pharyngitis and tonsillitis) in our study is similar to rates reported for the United Kingdom (62%) (Hawker et al., 2014), the United States (60%) (Barnett and Linder, 2014), and Sweden (60%) (Tystrup et al., 2016). In this case, only the Netherlands had a lower rate (55%) (Hawker et al., 2014). Our findings for acute bronchitis suggest there is much room for improvement in this area, as 58.65% of patients presenting with this condition were prescribed antibiotics *versus* just 26.2% of those in Sweden (Tystrup et al., 2016) and 52.1% of those in the Netherlands (Van den Broek d’Obrenan et al., 2014). Differences in prescription rates could be due to structural or organizational differences, such as nurse triage processes or PHC physicians recording practices. In Sweden nurses’ triage is performed according to guidelines which in many cases avoid the need for patients to visit PHC physicians (Tystrup et al., 2016). As for electronic medical records (EMR), not all health care organizations use the same classification of diseases. We used ICD codes, but the existence of other options such as the Current Procedural Terminology (published by the American Medical Association) or Snomed (product of the College of American Pathologists), can introduce differences in disease codification. Finally, EMR is a physician dependent activity, Richard A. Young et al. published an observational study in which they found that PHC physicians spent more time in direct ambulatory patient care working in the EMR than they spent in face-to-face time with their patients, authors also recognized the existence of differences between time spending in EMR between American and European physicians (Young et al., 2018).

The findings of our intervention support previous reports that effective ASPs not only improve antibiotic use but can also be financially self-supporting (Dellit et al., 2007). In 2015 Dimitri M. Drekonja et al. evaluated the costs of effect of outpatient ASPs through a systematic review. The authors found only seven out of 50 studies in which dispensing costs were reported, significant cost reductions associated to ASPs were found in three, the authors admitted that drug costs were not universally reported (Drekonja et al., 2015). Future outpatient ASPs should include economic analysis in order to confirm the generalizability of our results.

Our study has some limitations. First, our data did not include information on antibiotic prescriptions issued in private practice; second, we were unable to identify which components of the ASP were the most effective; third, we did not have data on antibiotic therapy duration; fourth, as we did not cover a complete geographical region, we were unable to measure the impact of the intervention on the prevalence of antimicrobial resistance; and fifth, we did not have a comparison group.

We decided to perform a quasi-experimental study to avoid the risk of cross-contamination described in other behavioral intervention studies. In 2016, Stewardson et al. published a single center, cluster randomized trial to assess the effect of enhanced performance feedback and patient participation on hand hygiene. The improvement attributable to patient participation did not reach statistical significance and the authors recognized the existence of an unavoidable cross-contamination effect (Stewardson et al., 2016). As our PHC area forms part of a centralized health care structure in which all professionals receive the same training and have access to the same information, it would have been impossible to avoid cross-contamination.

Our findings add to the body of evidence on the effectiveness of ASPs in several respects. We have provided qualitative, quantitative, and economic indicators on the effectiveness of a multifaceted intervention targeting both adult and pediatric patients and ours is one of few studies in Spain to analyze the impact in nursing homes. Finally, we analyzed the sustainability of our program by measuring post-intervention effects.

## Conclusions

Through a multimodal intervention, we were able to reduce antibiotic consumption, increase the use of narrow-spectrum antibiotics, and significantly improve the use of antibiotics to treat the most prevalent infections seen in PHC. Further studies should examine the generalizability of these findings to other health care systems.

## Data Availability Statement

The data analyzed in this study was obtained from Servei Català de Salut (Catalonian Health Service), the following licenses/restrictions apply Organic Law 15/1999, de 13 de December, Protection of personal Data.BOENum. 298, 14th December de 1999, pages 43088 a 43099. Requests to access these datasets should be directed to Servei Català de Salut (Catalonian Health Service), atenciociutadana@catsalut.cat.

## Author Contributions

All authors contributed in the design of the antimicrobial stewardship interventions and in the development of local guidelines on antibiotic. PM-L, RM, and RT implemented the interventions in all primary health care centers. EC and LB acted as expert consultants on antibiotic use.

## Funding

The project received a grant within the “Mútua Terrassa Primary Care Research fellowship 2017 (BE0106)”. The funders had no role in the study design, data collection and interpretation, or the decision to submit the work for publication. This study was conducted as a quality program.

## Conflict of Interest

The authors declare that the research was conducted in the absence of any commercial or financial relationships that could be construed as a potential conflict of interest.

## References

[B1] Barnett M. L. Linder J. A. (2014). Antibiotic Prescribing to Adults with Sore Throat in the United States, 1997-2010. JAMA Intern. Med. 174, 138–140. 10.1001/jamainternmed.2013.11673 24091806 PMC4526245

[B2] Catálogo de Centros de Atención Primaria del SNS y de Atención Urgente Extrahospitalaria Madrid (Ministerio de Sanidad Consumo y Bienester Social). [Accessed 04 June 2019]. Available from: https://www.mscbs.gob.es/ciudadanos/prestaciones/centrosServiciosSNS/centrosSalud/home.htm.

[B3] Dellit T. H. Owens R. C. McGowan J. E. Jr Gerding D. N. Weinstein R. A. Burke J. P. (2007). Infectious Diseases Society of America and the Society for Healthcare Epidemiology of America guidelines for developing an institutional program to enhance antimicrobial stewardship. Clin. Infect. Dis. 44, 159–177. 10.1086/510393 17173212

[B4] Drekonja D. Filice G. Greer N. Olson A. MacDonald R. Rutks I. (2015). Antimicrobial Stewardship in Outpatient Settings: A Systematic Review. Infect. Control Hosp. Epidemiol. 36 (2), 142–152. 10.1017/ice.2014.41 25632996

[B5] European Centre for Disease Prevention and Control (2017). Annual Report of the European Antimicrobial Resistance Surveillance Network (EARS-Net) Stockholm: ECDC; 2018. [Internet] [Accessed 04 June 2019]. Available from: https://www.ecdc.europa.eu/sites/default/files/documents/AER_for_2016-AMC.pdf.

[B6] European Centre for Disease Prevention and Control (2017). Surveillance of antimicrobial resistance in Europe 2016 (Stockholm: ECDC: Annual Report of the European Antimicrobial Resistance Surveillance Network (EARS-Net)).

[B7] European Centre for Disease Prevention and Control (2018). Antimicrobial resistance surveillance in Europe 2017 (Stockholm: ECDC: Annual Report of the European Antimicrobial Resistance Surveillance Network (EARS-Net)). [Accessed 04 June 2019]. Available from: https://www.ecdc.europa.eu/sites/default/files/documents/AER_for_2017-antimicrobial-consumption.pdf.

[B8] European Centre for Disease Prevention and Control, European Medicines Agency (2009). The bacterial challenge: time to react (Stockholm, Sweden: European Medicines Agency).

[B9] Fine A. M. Nizet V. Mandl K. D. (2012). Large-scale validation of the Centor and McIsaac scores to predict group A streptococcal pharyngitis. Arch. Intern. Med. 172 (11), 847–852. 10.1001/archinternmed.2012.950 22566485 PMC3627733

[B10] Goossens H. Ferech M. Vander Stichele R. Elseviers M. (2005). Outpatient antibiotic use in Europe and association with resistance: a cross-national database study. Lancet, 365 (9459), 579–587. 10.1016/S0140-6736(05)17907-0 15708101

[B11] Grijalva C. G. Nuorti J. P. Griffin M. R. (2009). Antibiotic prescription rated for acute respiratory tract infections in US ambulatory settings. JAMA. 203 (7), 758–766. 10.1001/jama.2009.1163 PMC4818952 19690308

[B12] Hürlimann D. Limacher A. Schabel M. Zanetti G. Berger C. Mühlemann K. (2015). Improvement of antibiotic prescription in outpatient care: a cluster-randomized intervention study using a sentinel surveillance network of physicians. J. Antimicrob. Chemother. 70 (2), 602–608. 10.1093/jac/dku394 25326088

[B13] Hansen M. J. Carson P. J. Leedahl D. D. Leedahl N. D. (2018). Failure of a Best Practice Alert to Reduce Antibiotic Prescribing Rates for Acute Sinusitis Across an Integrated Health System in the Midwest. J. Manage Care Spec Pharm. 24 (2), 154–159. 10.18553/jmcp.2018.24.2.154 PMC10398131 29384025

[B14] Hawker J. I. Smith S. Smith G. E. Morbey R. Johnson A. P. Fleming D. M. (2014). Trends in antibiotic prescribing in primary care for clinical syndromes subject to national recommendations to reduce antibiotic resistance, UK 1995-2011: analysis of a large database of primary care consultations. J. Antimicrob Chemother. 69 (12), 3423–3430. 10.1093/jac/dku291 25091508

[B15] Hersh A. L. Shapiro D. J. Pavia A. T. Shah S. S. (2011). Antibiotic prescribing in ambulatory pediatrics in the United States. Pediatrics. 128 (6), 1053–1061. 10.1542/peds.2011-1337 22065263

[B16] Lemiengre M. B. Van Driel M. L. Merenstein D. Young J. De Sutter A. I. (2012). Antibiotics for clinically diagnosed acute rhinosinusitis in adults. Cochrane Database Syst Rev. (10), CD006089. 10.1002/14651858.CD006089.pub4 23076918

[B17] Meeker D. Jeffrey A. Craig R. Friedberg M. W. Persell S. D. Goldstein N. J. (2016). Effect of Behavioral Interventions on Inappropriate Antibiotic Prescribing Among Primary Care Practices A Randomized Clinical Trial. JAMA 315 (6), 562–570. 10.1001/jama.2016.0275 26864410 PMC6689234

[B18] Murshid M. A. Mohaidin Z. (2017). Models and theories of prescribing decisions: A review and suggested a new model. Pharm. Pract. Granada 15, 990. 10.18549/PharmPract.2017.02.990 28690701 PMC5499356

[B19] O’Neill J. (2016). Tackling Drug-Resistant Infections Globally: Final Report and Recommendations (London, UK: Review on Antimicrobial Resistance), p. 1–84. 10.1371/journal.pmed.1002130

[B20] Ranji S. R. Steinman M. A. Shojania K. Gonzales R. (2008). Interventions to reduce unnecessary antibiotic prescribing: a systematic review and quantitative analysis. Med. Care 46, 847–862. 10.1097/MLR.0b013e318178eabd 18665065

[B21] Real Decreto 177/2014, de 21 de marzo, por el que se regula el sistema de precios de referencia y de agrupaciones homogéneas de medicamentos en el Sistema Nacional de Salud, y determinados sistemas de información en materia de financiación y precios de los medicamentos y productos sanitarios. [Online]. [Accessed 04 February 2020]. Available from https://www.boe.es/boe/dias/2014/03/25/pdfs/BOE-A-2014-3189.pdf.

[B22] Sanchez G. V. Fleming-Dutra K. E. Roberts R. M. Hicks L. A. (2016). Core Elements of Outpatient Antibiotic Stewardship. MMWR Recomm Rep. 65 (No. RR-6), 1–12. 10.15585/mmwr.rr6506a1 27832047

[B23] Seppälä H. Klaukka T. Vuopio-Varkila J. Moutiala A. Helenius H. Lager K. (1997). The effect of changes in the consumption of Macrolide antibiotics on erythromycin resistance in group A streptococci in Finland. Finnish Study Group for Antimicrobial Resistance. N Engl. J. Med. 337, 441–446. 10.1056/NEJM199708143370701 9250845

[B24] Smith S. M. Fahey T. Smucny J. Becker L. A. (2017). Antibiotics for acute bronchitis. Cochrane Systematic Rev. 6 (6), CD000245. 10.1002/14651858.CD000245.pub4 PMC6481481 28626858

[B25] Stewardson A. J. Sax H. Gayet-ageron A. Touveneau S. Longtin Y. Zingg W. (2016). Enhanced performance feedback and patient participation to improve hand hygiene compliance of health-care workers in the setting of established multimodal promotion: a single-center, cluster randomized controlled trial. Lancet Infect. Dis. 16 (12), 1345–1355. 10.1016/S1473-3099(16)30256-0 27599874

[B26] Strategic Action Plan to reduce the risk of selection and dissemination of antibiotic resistance. [Accessed 04 June 2019]. Available from: https://www.aemps.gob.es/en/publicaciones/publica/plan-estrategico-antibioticos/v2/docs/plan-estrategico-antimicrobianos-AEMPS.pdf.

[B27] Susceptibility of Escherichia coli Isolates to 3rd gen. cephalosporins in Spain, Netherlands, Estonia, Latvia, 1998 – 2014. The European Surveillance System on 2016-07-20.

[B28] Swedish work on containment of antibiotic resistance (2014). Solna: Public Health Agency of Sweden. [Accessed 04 June 2019]. Available from: https://www.folkhalsomyndigheten.se/contentassets/dae82c7afd424a57b57ec81818793346/swedish-work-on-containment-of-antibiotic-resistance.pdf.

[B29] Swedres-Svarm(2018). Consumption of antibiotics and occurrence of antibiotic resistance in Sweden Public Health Agency of Sweden. [Accessed 04 June 2019]. Available from: https://old.sva.se/globalassets/redesign2011/pdf/om_sva/publikationer/swedres_svarm2018.pdf.

[B30] Tystrup M. Beckman A. Mölstad S. Engström S. Lannering C. Melander E. (2016). Reduction in antibiotic prescribing for respiratory tract infections in Swedish primary care- a retrospective study of electronic patient records. BMC Infect. Dis. 16, 709. 10.1186/s12879-016-2018-9 27887585 PMC5124268

[B31] Van den Broek d’Obrenan J. Verheij T. J. Numans M. E. Van der Velden A. W. (2014). Antibiotic use in Dutch primary care: relation between diagnosis, consultation and treatment. J. Antimicrob Chemother. 69 (6), 1701–1707. 10.1093/jac/dku005 24508898

[B32] Vellinga A. Galvin S. Duane S. Callan A. Bennett K. Cormican M. (2016). Intervention to improve the quality of antimicrobial prescribing for urinary tract infection: a cluster randomized trial. CMAJ 188 (2), 108–115. 10.1503/cmaj.150601 26573754 PMC4732960

[B33] Venekamp P. R. Sanders S. L. Glasziou P. P. Del Mar C. B. Rovers M. M. (2015). Antibiotics for acute otitis media in children. Cochrane Database Syst. Rev. 2015 (6), CD000219. 10.1002/14651858.CD000219.pub4 PMC7043305 26099233

[B34] World Health Organization (2014). Antimicrobial resistance: global report on surveillance (Geneva, Switzerland: World Health Organization).

[B35] Young R. A. Burge S. K. Kumar K. A. Wilson J. M. Ortiz D. F. (2018). A Time-Motion Study of Primary Care Physicians’ Work in the Electronic Health Record Era. Fam Med. 50 (2), 91–99. 10.22454/FamMed.2018.184803 29432623

