# Supplementary material for: Impact of a Multifaceted Antimicrobial Stewardship Intervention in a Primary Health Care Area: A Quasi-Experimental Study
Source: Front Pharmacol. 2020 Apr 2;11:398. doi: 10.3389/fphar.2020.00398 (PMC7142248; doi:10.3389/fphar.2020.00398)
Supplement: Supplementary file 1 [file DataSheet_1.pdf]

Supplementary Material

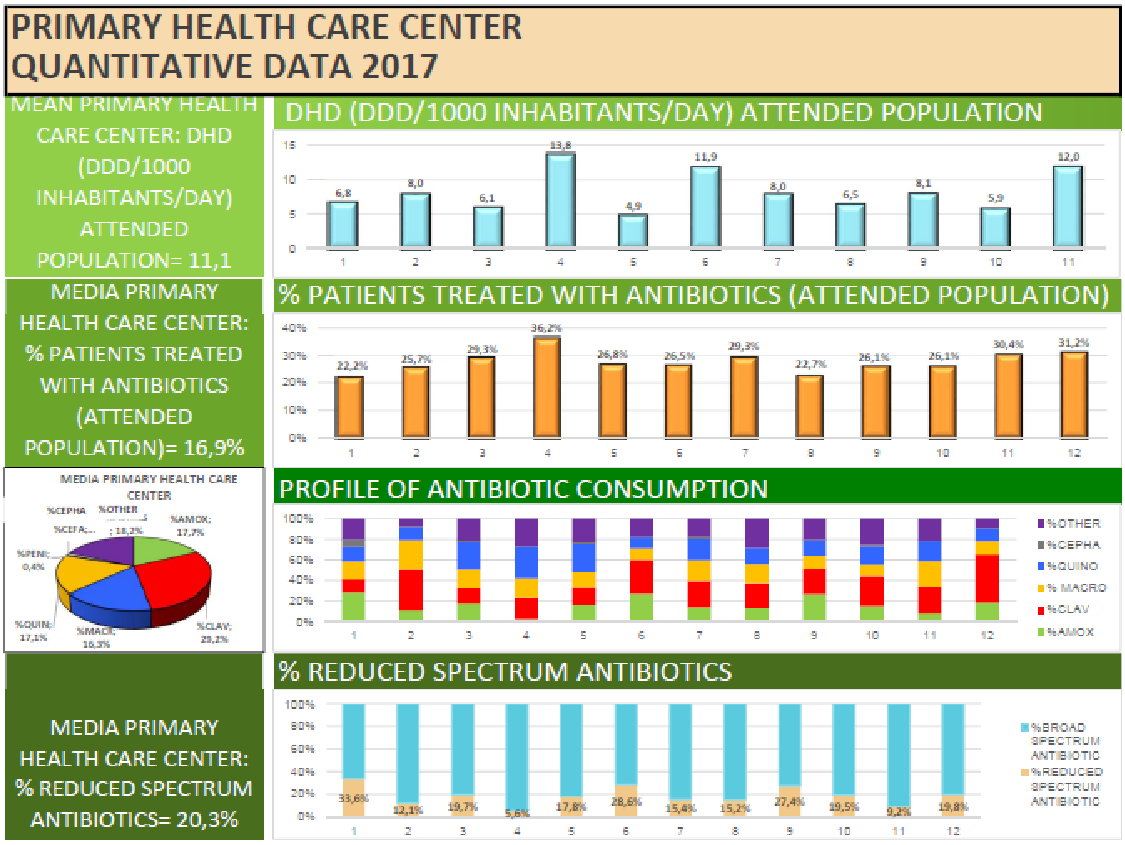

Figure 1: Sample personal feedback report sent to participating physicians every 3 months. Each number represents a prescribing physician. All physicians were informed of their number in a private message to be able to compare their performance with that of their anonymous colleagues and the center overall.

**Primary Outcome**
Defined daily dose per 1000 inhabitants per day (DID)
**Secondary Outcome**
a) (%) DID of narrow-spectrum antibiotics (ATC groups: J01CA. J01CE. J01CF. J01XX01) /DID of total antibiotics (J01)
b) DID of amoxicillin. amoxicillin/clavulanic acid. macrolides. and quinolones
c) Patients treated with antibiotic in the most prevalent outpatient syndromes:
**i) Pharyngotonsillitis (PT)**
(%) Number of patients diagnosed with PT and treated with antibiotics / Number patients diagnosed with PT
(%)Number of patients diagnosed with PT and treated with penicillin / Number patients treated with antibiotic and with PT diagnose
(%) Number of patients diagnosed with PT and treated with Amoxicillin / Number patients treated with antibiotic and with PT diagnose
(%) Number of patients diagnosed with PT and treated with Amoxicillin/Clavulanic and diagnosed of PT/ Number patients treated with antibiotic and with PT diagnose
(%) Number of patients diagnosed with PT and treated with Macrolides / Number patients treated with antibiotic and with PT diagnose
**ii) Acute Otitis Media (AOM)**
(%) Number of patients diagnosed with AOM and treated with antibiotic / Number patients with AOM diagnose.
(%) Number of patients diagnosed with AOM and treated with Amoxicillin / Number patients treated with antibiotic and with AOM diagnose
(%) Number of patients diagnosed with AOM and treated with Amoxicillin/Clavulanic / Number patients treated with antibiotic and with AOM diagnose
(%) Number of patients diagnosed with AOM and treated with Macrolides / Number patients treated with antibiotic and with AOM diagnose
(%) Number of patients diagnosed with AOM and treated with antibiotic excluding Macrolides. Amoxicillin or Amoxicillin/Clavulanic/ Number patients treated with antibiotic and with AOM diagnose
**iii) Urinary Tract Infection (UTI)**
(%) Number of patients diagnosed with UTI and treated with antibiotic / Number patients with UTI diagnose.
(%) Number of patients diagnosed with UTI and treated with Fosfomycin / Number patients treated with antibiotic and with UTI diagnose
(%) Number of patients diagnosed with UTI and treated with Amoxicillin/Clavulanic / Number patients treated with antibiotic and with UTI diagnose
(%) Number of patients diagnosed with UTI and treated with Quinolones / Number patients treated with antibiotic and with UTI diagnose
**iv) Acute Sinusitis (ASin)**
(%) Number of patients diagnosed with ASin and treated with antibiotic / Number patients with AS diagnose.
**v) Acute Bronchitis (AB)**
(%) Number of patients diagnosed with AB and treated with antibiotic / Number patients with AB diagnose.
d) Costs associated to antimicrobial consumption. ATC code J01 (Euros)

e)	(%) Population supplied with at least one antibiotic during the study periods/Total population attending the primary health care area.
f)	Antibiotic consumption in private geriatric homes measured in Daily Define Dose per 100 bed days.

Table 1: Primary and secondary outcome indicators

	Adults					Children (0-14 years)				
	2016	2017	2018	2016/2018 Difference	Statistical Analysis	2016	2017	2018	2016/2018 Difference	Statistical Analysis
**Patients treated with antibiotics, %**	70.94%	71.47%	71.40%	0.46%	p=0.69	51.15%	54.83%	49.28%	-1.87%	p=0.51
**Patients treated with fosfomycin, %**	39.58%	43.22%	45.36%	5.78%		7.46%	7.86%	13.99%	6.53%	
**Patients treated with amoxicillin/clavulanic acid, %**	6.15%	5.24%	4.24%	-1.95%		62.44%	47.97%	29.45%	-32.99%	
**Patients treated with quinolones, %**	27.52%	23.42%	20.47%	-7.05%	p<0.05	N/A	N/A	N/A	N/A	p<0.05
**Patients treated with cefuroxime, %**	26.76%	28.12%	29.93%	3.17%		6.72%	15.18%	21.87%	15.15%	
**Patients treated with other antibiotics, %**	N/A	N/A	N/A	N/A		23.38%	29.00%	34.69%	-11.31%	

Table 2: Percentage of patients treated with antibiotic and antibiotic choice for the treatment of urinary tract infection

N/A: Does Not Apply

	Adults					Children (0-14 years)				
	2016	2017	2018	2016/2018 Difference	Statistical Analysis	2016	2017	2018	2016/2018 Difference	Statistical Analysis
**Patients diagnosed with Acute Sinusitis and treated with antibiotics, %**	77.50%	74.99%	68.12%	-9.38%	P<0.05	80.64%	82.48%	81.27%	0.64%	P=0.37
**Patients diagnosed with Acute Bronchitis and treated with antibiotics, %**	70.06%	65.18%	58.65%	-11.42%	P<0.05	29.19%	27.05%	22.78%	-6.41%	P<0.05

Table 3: Percentage of patients treated with antibiotic with diagnosis of Acute Sinusitis and Acute Bronchitis

	2016	2017	2018	2016/2018 Difference
**Total Antibiotic Spending**	905,700.76	865,630.48	793,765.89	-111,934.87
**Amoxicillin (J01CA04)**	104,627.02	104,604.57	93,728.93	-108,98.09
**Amoxicillin/clavulanic (J01CR02)**	234,707.94	214,288.21	188,772.18	-45,935.76
**Macrolides (J01FA)**	185,384.74	176,483.27	15,3203.62	-32,181.12
**Erythromycin (J01FA01)**	1,496.08	1,145.48	590.6	-905.48
**Spiramycin (J01FA02)**	273.78	207.09	210.6	-63.18
**Josamycin (J01FA07)**	2,111.11	2,213.52	1,263.07	-848.04
**Clarithromycin (J01FA09)**	34,059.79	28,562.9	26,338.00	-7,721.79
**Azithromycin (J01FA10)**	14,5730.65	142,568.51	12,3658.63	-22,072.02
**Quinolones (J01M)**	200,781.19	174,542.56	15,7306.02	-43,475.17
**Ciprofloxacin (J01MA02)**	17,613.79	16,151.91	13,780.71	-3,833.08
**Norfloxacin (J01MA06)**	8,961.73	6,981.65	5,071.62	-3,890.11
**Levofloxacin (J01MA12)**	144,907.61	133,235.82	127,482.71	-17,424.90
**Moxifloxacin (J01MA14)**	29,227.62	18,114.48	10,947.5	-18,280.12
**Fosfomycin (J01XX01)**	53,056.71	54,851.47	54,595.91	1,539.20

Table 4: Economic analysis (euros) per year by generic names and antibiotic groups

frontiers

Figure 2: Local guideline for adult patients

MútuaTerrassa
ASSISTENCIAL
Atenció Primària

PROA

Programa d'Optimització de l'ús d'Antibiòtics

Tractament empíric de les malalties infeccioses en **adults**

- Per a un tractament antibiòtic empíric més racional s'han de considerar uns principis bàsics:
  - Cal conèixer els microorganismes més probables de cada infecció i utilitzar antibiòtics només si hi ha sospita d'un procés d'origen bacterià, si és factible, amb el suport de proves de diagnòstic ràpid.
  - En el moment de la indicació de l'antibiòtic cal valorar la seva eficàcia (espectre antibacterià), resistències, toxicitat, efectes secundaris, comoditat posològica, durada del tractament i preu.

1-205014 | 05/ 2017 | Id: 8258

INFECCIONS RESPIRATÒRIES

SINUSITIS AGUDA
- Iniciar tractament en pacients amb clínica lleugera i persistent de més de 10 dies o greu de 3-4 dies: Amoxicil·lina VO 750-1.000 mg / 8 h durant 7 dies.
- Si no millora a les 48 h per sospita de resistència als betalactàmics o bé infecció per Haemophilus: Ampliar l'espectre substituint per amoxicil·lina-clavulànic VO 875/125 mg / 8 h durant 7 dies.
- Al·lèrgics a la penicil·lina o intolerants: Levofloxacina VO 500 mg / 24 h durant 7 dies.

FARINGOAMIGDALITIS AGUDA
- Penicil·lina VO 500 mg / 12 h o amoxicil·lina 500 mg / 8 h durant 7 dies.
- En cas d'al·lèrgia confirmada a la penicil·lina: Es recomana clindamicina VO 300 mg / 8 h o bé macròlid de 16 àtoms (josamicina VO 500 mg / 12 h durant 7 dies).

OTITIS MITJANA AGUDA (OMA)
- Si no hi ha signes de gravetat ni factors de risc, no es recomana tractament antibiòtic.
- Si no millora en 48-72 h: Amoxicil·lina VO 500 mg / 8 h durant 7 dies i reavaluar en 48 h.
- Si no millora: Substituir per amoxicil·lina-clavulànic VO 875/125 mg / 8 h durant 7 dies.
- Al·lèrgics als betalactàmics: Levofloxacina VO 500 mg / 24 h durant 7 dies.

OTITIS EXTERNA
- Otitis externa difusa: Ciprofloxacina tòpica 0,3% 2-4 gotes / 12 h durant 7 dies.
- Otitis externa circumscrita (furóncol): Fusídic cada 8 h durant 7-10 dies. Si hi ha més afectació de teixits (cel·lulitis): Amoxicil·lina-clavulànic VO 875/125 mg / 8 h durant 7 dies.
- Al·lèrgics als betalactàmics: Clindamicina VO 300 mg / 8 h durant 7 dies.
- En cas d'otomicosi o miringitis ampul·lar lleu no es recomana tractament antibiòtic.
- En cas de miringitis ampul·lar extensa (sospita de Mycoplasma): Azitromicina VO 500 mg / 24 h durant 3 dies.

BRONQUITIS
- Més del 90% de l'etiologia és vírica amb procés autolimitat i no requereix tractament antibiòtic.
- Si hi ha sospita clínica d'infecció per B. pertussis: Iniciar tractament amb Azitromicina VO 500 mg/dia el 1r dia i 250 mg/dia del 2n al 5è dia.

PROA adults

BRONQUIÈCTASIS SOBREINFECTADES
- Infecció aguda sense exacerbacions: Amoxicil·lina-clavulànic VO 875/125 mg / 8 h durant 7 dies.
- Infecció aguda amb > 2 exacerbacions a l'any: Levofloxacina VO 500 mg/ 24 h durant 10 dies.

EXACERBACIÓ EN MPOC
- FEV1 > 50 % (moderat-lleu) en pacient sense comorbiditats (DM, cirrosi hepàtica, IRC, cardiopatia isquèmica o insuficiència cardíaca): Amoxicil·lina-clavulànic VO 875/125 mg / 8 h durant 7 dies.
- Al·lèrgics a betalactàmics: Levofloxacina VO 500 mg / 24 h durant 7 dies.
- FEV1 > 50% (moderat-lleu) amb comorbiditats o > 2 exacerbacions a l'any: Amoxicil·lina-clavulànic VO 875/125 mg / 8 h durant 7 dies o Levofloxacina VO 500 mg / 24 h durant 7 dies.
- FEV1 < 50 % (greu) sense risc de Pseudomonas aeruginosa: Amoxicil·lina-clavulànic VO 875/125 mg / 8 h durant 7 dies o Levofloxacina VO 500 mg / 24 h durant 7 dies.
- FEV1 < 50 % (greu) amb Pseudomonas aeruginosa: Levofloxacina VO 500 mg / 24 h durant 10 dies.

PNEUMÒNIA ADQUIRIDA A LA COMUNITAT (PNAC)
PNAC amb forma de presentació típica:
- Pacient < 65 anys sense patologia de base: Amoxicil·lina VO 1 g / 8 h durant 7 dies i reavaluar en 48-72 h. Si no respon, substituir per Amoxicil·lina-clavulànic VO 875/125 mg / 8 h durant 7 dies. En cas d'al·lèrgia: Levofloxacina VO 500 mg / 24 h durant 7 dies.
- Pacient > 65 anys o amb malaltia crònica: Amoxicil·lina-clavulànic VO 875/125 mg / 8 h durant 7 dies. En cas d'al·lèrgia: Levofloxacina VO 500 mg / 24 h durant 7 dies.

PNAC amb forma de presentació atípica:
- Claritromicina VO 500 mg / 12 h durant 7 dies o Azitromicina VO 500 mg / 24 h durant 3 dies.

PNAC amb forma de presentació indeterminada:
- Levofloxacina VO 500 mg / 24 h durant 7 dies. Al·lèrgics o alternativa: Amoxicil·lina-clavulànic VO 875/125 mg + Claritromicina VO 500 mg / 12 h durant 7 dies.

INFECCIONS GENITOURINÀRIES

CISTITIS NO COMPLICADA EN LA DONA
- Elecció: Fosfomicina trometamol VO 3 g, dosi única.
- Alternativa: Cefuroxima VO 250-500 mg / 12 h durant 3 dies o Ciprofloxacina VO 250-500 mg / 12 h durant 3 dies.

CISTITIS DE REPETICIÓ EN LA DONA
Reinfecció:
- < 3 episodis/any: Tractament igual que cistitis no complicada.
- ≥ 3 episodis/any: Tractament empíric Cefuroxima VO 500 mg / 12 h durant 7 dies, prèvia recollida d'orina per a urinocultiu. En aquests casos es recomana profilaxi amb dosis baixes d'antibiòtics durant 6 mesos. Si es relaciona amb el coit cal fer profilaxi postcoital amb una dosi única d'antibiòtic).

Recidiva: Tractament segons antibiograma, de 2 a 6 setmanes.

CISTITIS DE LA DONA GESTANT
- Requereix urinocultiu previ al tractament antibiòtic empíric. La cistitis i la bacteriúria asimptomàtica es tracten de la mateixa forma, i ens guiarem per l'antibiograma.
- Fosfomicina trometamol VO 3 g, dosi única o Cefuroxima axetil VO 250-500 mg / 12 h durant 5 dies.

PIELONEFRITIS AGUDA NO COMPLICADA EN LA DONA
- Requereix urinocultiu previ al tractament antibiòtic empíric.
- Empíric: Cefuroxima axetil VO 500 mg / 8 h durant 10 dies.
- Dirigit: Canviar a Ciprofloxacina VO 500 mg / 12 h durant 7 dies, en funció de l'antibiograma.

INFECCIÓ URINÀRIA EN L'HOME
- Les cistitis agudes es consideren complicades. Sospitar sempre pielonefritis subclínica o afectació renal complicada, per la qual cosa cal fer un urinocultiu previ al tractament.
- Les pautes curtes estan contraindicades.
- La recaiguda o infecció recurrent indica infecció crònica de la pròstata o anomalies funcionals o anatòmiques de la via urinària; requereixen tractament durant 4-6 setmanes i consulta a l'uròleg.
- El 50% dels homes amb infecció recurrent i el 90% dels que tenen febre tenen afectació de la pròstata que pot evolucionar a abscés de pròstata o prostatitis bacteriana crònica.
- Cal fer un estudi urològic després del segon episodi documentat d'infecció urinària.

CISTITIS I PIELONEFRITIS AGUDA
- Pielonefritis complicada: Derivar a l'hospital.
- Empíric: Cefuroxima axetil VO 500 mg / 8 h durant 14 dies.
- Dirigit: Canviar a Ciprofloxacina VO 500 mg / 12 h en funció de l'antibiograma.

PROSTATITIS
- Tractament ambulatori si el pacient no està tòxic, edat < 65 anys i no immunodepressió. Altrament, ingrés hospitalari.
- Urinocultiu i tractament antibiòtic precoç per evitar complicacions, ja que es tracta d'una malaltia greu.
- Mesures generals: repòs, hidratació, antipirètics, analgèsics i laxants.
- Prostatitis aguda no complicada: Cefuroxima axetil VO 500 mg / 8 h durant 28 dies.
- Les quinolones s'han d'utilitzar guiades per antibiograma: Ciprofloxacina VO 500 mg / 12 h durant 28 dies o Levofloxacina VO 500 mg / 24 h durant 28 dies o Ofloxacina VO 400 mg / 12 h durant 28 dies.
- Prostatitis crònica bacteriana: Allargar el tractament fins a 6-8 setmanes.
- Prostatitis complicada: Derivar a l'hospital.

INFECCIÓ DEL TRACTE URINARI (ITU) DEL PACIENT SONDAT
- Només es tractarà en cas d'ITU simptomàtica amb febre, dolor i tenesme vesical i recanvi de catèter. Cal fer el tractament durant 7 dies si no hi ha evidència de prostatitis o PNA, i durant 14 dies si n'hi ha.
- Tractament ambulatori: Canvi de la sonda urinària, urinocultiu i antibiograma.
- Profilaxi antibiòtica només en el canvi de sonda en pacients amb immunodepressió, traumatisme intraprocediment o antecedents d'infeccions prèvies en el context de recanvis anteriors.
- Remetre a l'hospital si hi ha signes de gravetat.
- Cefuroxima axetil VO 500 mg / 8 h durant 14 dies.
- Al·lèrgics: Ciprofloxacina VO 500 mg / 12 h durant 14 dies (però cal estar alerta al fracàs per risc de resistències).

BACTERIÚRIA ASIMPTOMÀTICA
Es recomana el cribratge i tractament de la bacteriúria asimptomàtica només en cas de dones embarassades i abans d'una intervenció genitourinària invasiva en la qual hi hagi risc d'hemorràgia mucosa.
- Fosfomicina trometamol VO 3 g, dosi única.
- Alternatives: Amoxicil·lina-clavulànic VO 500/125 mg / 8 h durant 5 dies o Cefixima VO 400 mg / 24 h durant 5 dies o Nitrofurantoïna VO 50-100 mg / 6 h durant 7 dies.

URETRITIS I CERVICITIS
- Ceftriaxona IM 250 mg, dosi única + Azitromicina VO 1 g, dosi única.
- Al·lèrgics als betalactàmics: es pot substituir la Ceftriaxona per Ciprofloxacina VO 500 mg, dosi única. L'alternativa a l'Azitromicina és la Doxiciclina VO 100 mg / 12 h durant 7 dies.

VULVOVAGINITIS
Tricomonosi:
- Metronidazol VO 2 g, dosi única. Alternativa: Tinidazol VO 2g, dosi única.

Vaginosi bacteriana:
- No complicada: Metronidazol vaginal (gel 0,75%) 1 aplicació/dia, a la nit, durant 5 dies. Alternativa: Metronidazol VO 500 mg / 8 h durant 7 dies. Al·lèrgics al Metronidazol: Clindamicina vaginal (crema 2%) 5 g, a la nit, durant 7 dies.
- No complicada en l'embaràs: Metronidazol VO 250 mg / 8 h durant 7 dies.

Candidiasi:
- No complicada: Clotrimazol vaginal (comprimits) VO 500 mg, dosi única. Alternativa: Fluconazol VO 150 mg, dosi única.
- No complicada en l'embaràs: Clotrimazol vaginal (crema 2%) 5 g durant 7 dies.

INFECCIONS DE LA PELL
- Com a norma general, cal cobertura de S. aureus i S. pyogenes.
- En infeccions superficials, poc extenses, pot fer-se servir un antibiòtic tòpic com retapamulina o àcid fusídic.

CEL·LULITIS
- Amoxicil·lina-clavulànic VO 875/125 mg / 8 h durant 7 dies.
- Al·lèrgics: Clindamicina VO 600 mg / 8 h durant 7 dies.

IMPETIGEN
- Tòpic: Fusídic cada 8 h durant 7-10 dies.
- Oral: Amoxicil·lina-clavulànic VO 500/125 mg / 8 h durant 7-10 dies. Alternativa: Clindamicina VO 300 mg / 8 h durant 7-10 dies.
- Impetigen no ampul·lar: s'acostuma a resoldre espontàniament sense tractament i sense cicatriu en 2 setmanes. El tractament accelera la seva resolució. En cas d'impetigen de repetició, la descolonització nasal amb antibiòtics tòpics (Mupirocina, 3 cops/dia durant 2-5 dies) pot disminuir-ne la freqüència.

FOL·LICULITIS (S. aureus)
- El tractament dels factors predisponents pot resoldre la situació: Falta d'higiene, humitat, oclusió, saunes (P. aeruginosa, càndida) o ús perllongat d'antibiòtics (acne). En Fol·liculitis o Furunculosis de repetició, la descolonització tòpica nasal pot disminuir-ne la freqüència.

MOSSEGADES
- Amoxicil·lina-clavulànic VO 500/125 mg / 8 h durant 5 dies.
- Alternativa: Levofloxacina VO 500 mg / 24 h + Clindamicina VO 300 mg / 8 h durant 5 dies.

INFECCIONS DENTALS

ABSCÉS PERIAPICAL
- Amoxicil·lina-clavulànic VO 875/125 mg / 8 h durant 7 dies.
- Al·lèrgic als betalactàmics: Clindamicina VO 300 mg / 6 h durant 7 dies o Azitromicina VO 500 mg / 24 h durant 3 dies.

Figure 3: Local guideline for pediatric patients

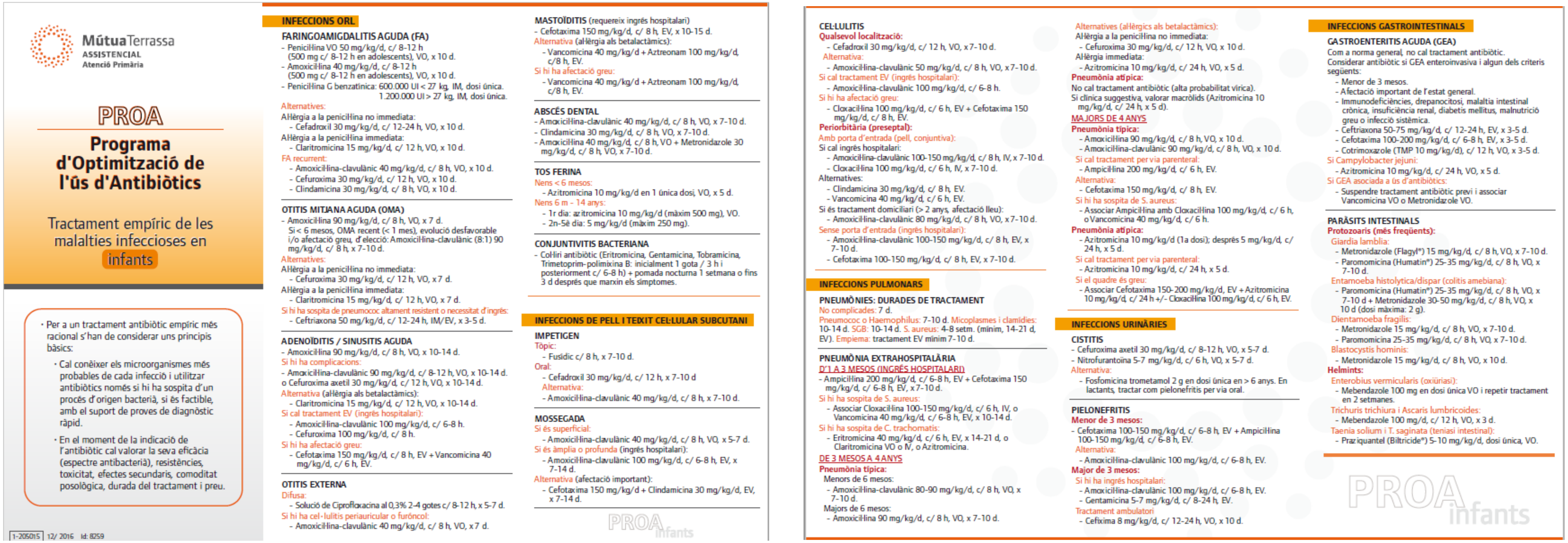

MútuaTerrassa
ASSISTENCIAL
Atenció Primària

PROA

Programa d'Optimització de l'ús d'Antibiòtics

Tractament empíric de les malalties infeccioses en infants

- Per a un tractament antibiòtic empíric més racional s'han de considerar uns principis bàsics:
  - Cal conèixer els microorganismes més probables de cada infecció i utilitzar antibiòtics només si hi ha sospita d'un procés d'origen bacterià, si és factible, amb el suport de proves de diagnòstic ràpid.
  - En el moment de la indicació de l'antibiòtic cal valorar la seva eficàcia (espectre antibacterià), resistències, toxicitat, efectes secundaris, comoditat posològica, durada del tractament i preu.

1-205015 12/ 2016 Id: 8259

INFECCIONS ORL

FARINGOAMIGDALITIS AGUDA (FA)
- Penicil·lina VO 50 mg/kg/d, c/ 8-12 h (500 mg c/ 8-12 h en adolescents), VO, x 10 d.
- Amoxicil·lina 40 mg/kg/d, c/ 8-12 h (500 mg c/ 8-12 h en adolescents), VO, x 10 d.
- Penicil·lina G benzatínica: 600.000 UI < 27 kg, IM, dosi única. 1.200.000 UI > 27 kg, IM, dosi única.

Alternatives:
Al·lèrgia a la penicil·lina no immediata:
- Cefadroxil 30 mg/kg/d, c/ 12-24 h, VO, x 10 d.

Al·lèrgia a la penicil·lina immediata:
- Claritromicina 15 mg/kg/d, c/ 12 h, VO, x 10 d.

FA recurrent:
- Amoxicil·lina-clavulànic 40 mg/kg/d, c/ 8 h, VO, x 10 d.
- Cefuroxima 30 mg/kg/d, c/ 12 h, VO, x 10 d.
- Clindamicina 30 mg/kg/d, c/ 8 h, VO, x 10 d.

OTITIS MITJANA AGUDA (OMA)
- Amoxicil·lina 90 mg/kg/d, c/ 8 h, VO, x 7 d.
  Si < 6 mesos, OMA recent (< 1 mes), evolució desfavorable i/o afectació greu, d'elecció: Amoxicil·lina-clavulànic (8:1) 90 mg/kg/d, c/ 8 h, x 7-10 d.

Alternatives:
Al·lèrgia a la penicil·lina no immediata:
- Cefuroxima 30 mg/kg/d, c/ 12 h, VO, x 7 d.

Al·lèrgia a la penicil·lina immediata:
- Claritromicina 15 mg/kg/d, c/ 12 h, VO, x 7 d.

Si hi ha sospita de pneumococ altament resistent o necessitat d'ingrés:
- Ceftriaxona 50 mg/kg/d, c/ 12-24 h, IM/EV, x 3-5 d.

ADENOÏDITIS / SINUSITIS AGUDA
- Amoxicil·lina 90 mg/kg/d, c/ 8 h, VO, x 10-14 d.

Si hi ha complicacions:
- Amoxicil·lina-clavulànic 90 mg/kg/d, c/ 8-12 h, VO, x 10-14 d. o Cefuroxima axetil 30 mg/kg/d, c/ 12 h, VO, x 10-14 d.

Alternativa (al·lèrgia als betalactàmics):
- Claritromicina 15 mg/kg/d, c/ 12 h, VO, x 10-14 d.

Si cal tractament EV (ingrés hospitalari):
- Amoxicil·lina-clavulànic 100 mg/kg/d, c/ 6-8 h.
- Cefuroxima 100 mg/kg/d, c/ 8 h.

Si hi ha afectació greu:
- Cefotaxima 150 mg/kg/d, c/ 8 h, EV + Vancomicina 40 mg/kg/d, c/ 6 h, EV.

OTITIS EXTERNA
Difusa:
- Solució de Ciprofloxacina al 0,3% 2-4 gotes c/ 8-12 h, x 5-7 d.

Si hi ha cel·lulitis periauricular o furóncol:
- Amoxicil·lina-clavulànic 40 mg/kg/d, c/ 8 h, VO, x 7 d.

MASTOÏDITIS (requereix ingrés hospitalari)
- Cefotaxima 150 mg/kg/d, c/ 8 h, EV, x 10-15 d.

Alternativa (al·lèrgia als betalactàmics):
- Vancomicina 40 mg/kg/d + Aztreonam 100 mg/kg/d, c/8 h, EV.

Si hi ha afectació greu:
- Vancomicina 40 mg/kg/d + Aztreonam 100 mg/kg/d, c/8 h, EV.

ABSCÉS DENTAL
- Amoxicil·lina-clavulànic 40 mg/kg/d, c/ 8 h, VO, x 7-10 d.
- Clindamicina 30 mg/kg/d, c/ 8 h, VO, x 7-10 d.
- Amoxicil·lina 40 mg/kg/d, c/ 8 h, VO + Metronidazole 30 mg/kg/d, c/ 8 h, VO, x 7-10 d.

TOS FERINA
Nens < 6 mesos:
- Azitromicina 10 mg/kg/d en 1 única dosi, VO, x 5 d.

Nens 6 m - 14 anys:
- 1r dia: azitromicina 10 mg/kg/d (màxim 500 mg), VO.
- 2n-5è dia: 5 mg/kg/d (màxim 250 mg).

CONJUNTIVITIS BACTERIANA
- Col·liri antibiòtic (Eritromicina, Gentamicina, Tobramicina, Trimetoprim-polimixina B: inicialment 1 gota / 3 h i posteriorment c/ 6-8 h) + pomada nocturna 1 setmana o fins 3 d després que marxin els símptomes.

INFECCIONS DE PELL I TEIXIT CEL·LULAR SUBCUTANI

IMPETIGEN
Tòpic:
- Fusídic c/ 8 h, x 7-10 d.

Oral:
- Cefadroxil 30 mg/kg/d, c/ 12 h, x 7-10 d

Alternativa:
- Amoxicil·lina-clavulànic 40 mg/kg/d, c/ 8 h, x 7-10 d.

MOSSEGADA
Si és superficial:
- Amoxicil·lina-clavulànic 40 mg/kg/d, c/ 8 h, VO, x 5-7 d.

Si és àmplia o profunda (ingrés hospitalari):
- Amoxicil·lina-clavulànic 100 mg/kg/d, c/ 6-8 h, EV, x 7-14 d.

Alternativa (afectació important):
- Cefotaxima 150 mg/kg/d + Clindamicina 30 mg/kg/d, EV, x 7-14 d.

PROA infants

CEL·LULITIS
Qualsevol localització:
- Cefadroxil 30 mg/kg/d, c/ 12 h, VO, x 7-10 d.

Alternativa:
- Amoxicil·lina-clavulànic 50 mg/kg/d, c/ 8 h, VO, x 7-10 d.

Si cal tractament EV (ingrés hospitalari):
- Amoxicil·lina-clavulànic 100 mg/kg/d, c/ 6-8 h.

Si hi ha afectació greu:
- Cloxacil·lina 100 mg/kg/d, c/ 6 h, EV + Cefotaxima 150 mg/kg/d, c/ 8 h, EV.

Periorbitària (preseptal):
Amb porta d'entrada (pell, conjuntiva):
Si cal ingrés hospitalari:
- Amoxicil·lina-clavulànic 100-150 mg/kg/d, c/ 8 h, IV, x 7-10 d.
- Cloxacil·lina 100 mg/kg/d, c/ 6 h, IV, x 7-10 d.

Alternatives:
- Clindamicina 30 mg/kg/d, c/ 8 h, EV.
- Vancomicina 40 mg/kg/d, c/ 6 h, EV.

Si és tractament domiciliari (> 2 anys, afectació lleu):
- Amoxicil·lina-clavulànic 80 mg/kg/d, c/ 8 h, VO, x 7-10 d.

Sense porta d'entrada (ingrés hospitalari):
- Amoxicil·lina-clavulànic 100-150 mg/kg/d, c/ 8 h, EV, x 7-10 d.
- Cefotaxima 100-150 mg/kg/d, c/ 8 h, EV, x 7-10 d.

INFECCIONS PULMONARS

PNEUMÒNIES: DURADES DE TRACTAMENT
No complicades: 7 d.
Pneumococ o Haemophilus: 7-10 d. Micoplasmes i clamídies: 10-14 d. SGB: 10-14 d. S. aureus: 4-8 setm. (mínim, 14-21 d, EV). Empiema: tractament EV mínim 7-10 d.

PNEUMÒNIA EXTRAHOSPITALÀRIA
D'1 A 3 MESOS (INGRÉS HOSPITALARI)
- Ampicil·lina 200 mg/kg/d, c/ 6-8 h, EV + Cefotaxima 150 mg/kg/d, c/ 6-8 h, EV, x 7-10 d.

Si hi ha sospita de S. aureus:
- Associar Cloxacil·lina 100-150 mg/kg/d, c/ 6 h, IV, o Vancomicina 40 mg/kg/d, c/ 6-8 h, EV, x 10-14 d.

Si hi ha sospita de C. trachomatis:
- Eritromicina 40 mg/kg/d, c/ 6 h, EV, x 14-21 d, o Claritromicina VO o IV, o Azitromicina.

DE 3 MESOS A 4 ANYS
Pneumònia típica:
Menors de 6 mesos:
- Amoxicil·lina-clavulànic 80-90 mg/kg/d, c/ 8 h, VO, x 7-10 d.

Majors de 6 mesos:
- Amoxicil·lina 90 mg/kg/d, c/ 8 h, VO, x 7-10 d.

Alternatives (al·lèrgics als betalactàmics):
Al·lèrgia a la penicil·lina no immediata:
- Cefuroxima 30 mg/kg/d, c/ 12 h, VO, x 10 d.

Al·lèrgia immediata:
- Azitromicina 10 mg/kg/d, c/ 24 h, VO, x 5 d.

Pneumònia atípica:
No cal tractament antibiòtic (alta probabilitat vírica).
Si clínica suggestiva, valorar macròlids (Azitromicina 10 mg/kg/d, c/ 24 h, x 5 d).

MAJORS DE 4 ANYS
Pneumònia típica:
- Amoxicil·lina 90 mg/kg/d, c/ 8 h, VO, x 10 d.
- Amoxicil·lina-clavulànic 90 mg/kg/d, c/ 8 h, VO, x 10 d.

Si cal tractament per via parenteral:
- Ampicil·lina 200 mg/kg/d, c/ 6 h, EV.

Alternativa:
- Cefotaxima 150 mg/kg/d, c/ 8 h, EV.

Si hi ha sospita de S. aureus:
- Associar Ampicil·lina amb Cloxacil·lina 100 mg/kg/d, c/ 6 h, o Vancomicina 40 mg/kg/d, c/ 6 h.

Pneumònia atípica:
- Azitromicina 10 mg/kg/d (1a dosi); després 5 mg/kg/d, c/ 24 h, x 5 d.

Si cal tractament per via parenteral:
- Azitromicina 10 mg/kg/d, c/ 24 h, x 5 d.

Si el quadre és greu:
- Associar Cefotaxima 150-200 mg/kg/d, EV + Azitromicina 10 mg/kg/d, c/ 24 h +/- Cloxacil·lina 100 mg/kg/d, c/ 6 h, EV.

INFECCIONS URINÀRIES

CISTITIS
- Cefuroxima axetil 30 mg/kg/d, c/ 8-12 h, VO, x 5-7 d.
- Nitrofurantoïna 5-7 mg/kg/d, c/ 6 h, VO, x 5-7 d.

Alternativa:
- Fosfomicina trometamol 2 g en dosi única en > 6 anys. En lactants, tractar com pielonefritis per via oral.

PIELONEFRITIS
Menor de 3 mesos:
- Cefotaxima 100-150 mg/kg/d, c/ 6-8 h, EV + Ampicil·lina 100-150 mg/kg/d, c/ 6-8 h, EV.

Alternativa:
- Amoxicil·lina-clavulànic 100 mg/kg/d, c/ 6-8 h, EV.

Major de 3 mesos:
Si hi ha ingrés hospitalari:
- Amoxicil·lina-clavulànic 100 mg/kg/d, c/ 6-8 h, EV.
- Gentamicina 5-7 mg/kg/d, c/ 8-24 h, EV.

Tractament ambulatori
- Cefixima 8 mg/kg/d, c/ 12-24 h, VO, x 10 d.

INFECCIONS GASTROINTESTINALS

GASTROENTERITIS AGUDA (GEA)
Com a norma general, no cal tractament antibiòtic.
Considerar antibiòtic si GEA enteroinvasiva i algun dels criteris següents:
- Menor de 3 mesos.
- Afectació important de l'estat general.
- Immunodeficiències, drepanocitosi, malaltia intestinal crònica, insuficiència renal, diabetis mellitus, malnutrició greu o infecció sistèmica.
- Ceftriaxona 50-75 mg/kg/d, c/ 12-24 h, EV, x 3-5 d.
- Cefotaxima 100-200 mg/kg/d, c/ 6-8 h, EV, x 3-5 d.
- Cotrimoxazole (TMP 10 mg/kg/d), c/ 12 h, VO, x 3-5 d.

Si Campylobacter jejuni:
- Azitromicina 10 mg/kg/d, c/ 24 h, VO, x 5 d.

Si GEA asociada a ús d'antibiòtics:
- Suspendre tractament antibiòtic previ i associar Vancomicina VO o Metronidazole VO.

PARÀSITS INTESTINALS
Protozoaris (més freqüents):
Giardia lamblia:
- Metronidazole (Flagyl®) 15 mg/kg/d, c/ 8 h, VO, x 7-10 d.
- Paromomicina (Humatin®) 25-35 mg/kg/d, c/ 8 h, VO, x 7-10 d.

Entamoeba histolytica/dispar (colitis amebiana):
- Paromomicina (Humatin®) 25-35 mg/kg/d, c/ 8 h, VO, x 7-10 d + Metronidazole 30-50 mg/kg/d, c/ 8 h, VO, x 10 d (dosi màxima: 2 g).

Dientamoeba fragilis:
- Metronidazole 15 mg/kg/d, c/ 8 h, VO, x 7-10 d.
- Paromomicina 25-35 mg/kg/d, c/ 8 h, VO, x 7-10 d.

Blastocystis hominis:
- Metronidazole 15 mg/kg/d, c/ 8 h, VO, x 10 d.

Helmints:
Enterobius vermicularis (oxiüriasi):
- Mebendazole 100 mg en dosi única VO i repetir tractament en 2 setmanes.

Trichuris trichiura i Ascaris lumbricoides:
- Mebendazole 100 mg/d, c/ 12 h, VO, x 3 d.

Taenia solium i T. saginata (teniasi intestinal):
- Praziquantel (Biltricide®) 5-10 mg/kg/d, dosi única, VO.

PROA infants
